# Neutrophils in Psoriasis

**DOI:** 10.3389/fimmu.2019.02376

**Published:** 2019-10-09

**Authors:** Chih-Chao Chiang, Wei-Jen Cheng, Michal Korinek, Cheng-Yu Lin, Tsong-Long Hwang

**Affiliations:** ^1^Graduate Institute of Clinical Medical Sciences, College of Medicine, Chang Gung University, Taoyuan, Taiwan; ^2^Supervisor Board, Taoyuan Chinese Medicine Association, Taoyuan, Taiwan; ^3^Puxin Fengze Chinese Medicine Clinic, Taoyuan, Taiwan; ^4^School of Traditional Chinese Medicine, Chang Gung University, Taoyuan, Taiwan; ^5^Center for Traditional Chinese Medicine, Chang Gung Memorial Hospital, Taoyuan, Taiwan; ^6^Graduate Institute of Natural Products, College of Medicine, Chang Gung University, Taoyuan, Taiwan; ^7^Research Center for Chinese Herbal Medicine, Research Center for Food and Cosmetic Safety, and Graduate Institute of Health Industry Technology, Chang Gung University of Science and Technology, Taoyuan, Taiwan; ^8^Department of Biotechnology, College of Life Science, Kaohsiung Medical University, Kaohsiung, Taiwan; ^9^Chinese Herbal Medicine Research Team, Healthy Aging Research Center, Chang Gung University, Taoyuan, Taiwan; ^10^Department of Anesthesiology, Chang Gung Memorial Hospital, Taoyuan, Taiwan; ^11^Department of Chemical Engineering, Ming Chi University of Technology, New Taipei City, Taiwan

**Keywords:** neutrophils, psoriasis, immunity, respiratory burst, degranulation, neutrophil extracellular traps

## Abstract

Neutrophils are the most abundant innate immune cells. The pathogenic roles of neutrophils are related to chronic inflammation and autoimmune diseases. Psoriasis is a chronic systemic inflammatory disease affecting ~2–3% of the world population. The abundant presence of neutrophils in the psoriatic skin lesions serves as a typical histopathologic hallmark of psoriasis. Recent reports indicated that oxidative stress, granular components, and neutrophil extracellular traps from psoriatic neutrophils are related to the initial and maintenance phases of psoriasis. This review provides an overview on the recent (up to 2019) advances in understanding the role of neutrophils in the pathophysiology of psoriasis, including the effects of respiratory burst, degranulation, and neutrophil extracellular trap formation on psoriatic immunity and the clinical relationships.

## Introduction

**Figure 1:**
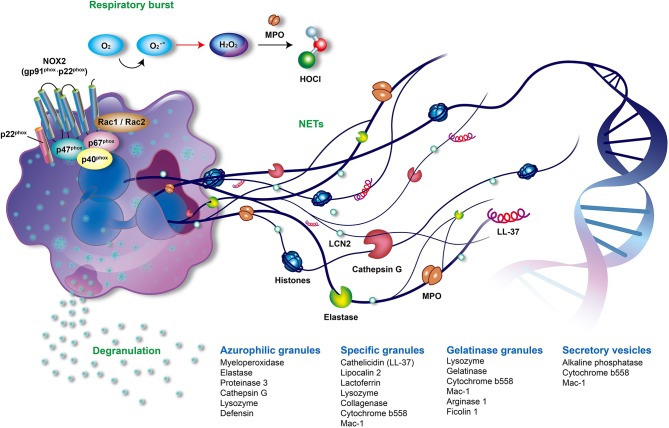
The offensive mechanisms of neutrophils in inflammation. In the activated neutrophils, respiratory burst, degranulation, and the formation of neutrophil extracellular traps (NETs) are the main mechanisms responsible for inflammation. During the respiratory burst, neutrophils utilize oxygen to produce reactive oxygen species (ROS) such as superoxide (${\text{O}}_{2}^{-}$), peroxide H_2_O_2_ or hypochlorous acid (HOCl) by activating NADPH oxidase (NOX2) and myeloperoxidase (MPO). As activation processes are triggered by diverse stimuli, neutrophils mobilize different granules including azurophilic granules, specific granules, gelatinase granules, and secretory vesicles. Subsequently, neutrophils degranulate and release various granular compounds of which neutrophil elastase (NE), MPO, proteinase 3, LL37, and lipocalin 2 play an important role in the pathogenesis of psoriasis. NETs are novel and pivotal components of neutrophils composed of extruded sticky and decondensed chromatin decorated with many antimicrobial compounds, such as histones, MPO, NE, and cathepsin G. The formation of NETs is involved in complicated inflammatory reactions and contributes to the pathogenesis of psoriasis. MPO, myeloperoxidase; NADPH, reduced nicotinamide adenine dinucleotide phosphate; NE, neutrophil elastase; NET, neutrophil extracellular trap; NOX2, NADPH oxidase.

Neutrophils are the most abundant cells in innate immunity. The main offensive functions of neutrophils include respiratory burst accompanied by reactive oxygen species (ROS) generation, degranulation (release of granules), and the formation of neutrophil extracellular traps (NETs) (Figure 1) (1, 2). Neutrophils shape adaptive immunity because they communicate and interact with the antigen-presenting cells and lymphocytes at the sites of inflammation (3, 4). Recently great attention was brought to the role of neutrophils in the development and progression of autoimmune diseases such as psoriasis.

Psoriatic neutrophils produce augmented respiratory burst with an overt accumulation of oxidative stress involving complicated inflammatory pathways. Proteases released in the degranulation step by neutrophils, such as myeloperoxidase (MPO), neutrophil elastase (NE), proteinase 3, and cathepsin G, participate in the generation of ROS, proteolytical activation of inflammatory mediators, and formation of autoantigens in psoriasis. The complexity and prevalence of psoriasis among the population since antiquity encouraged scientists to study the etiology of the disease and its relation to the immune system and inflammatory responses. Elegant reviews described the role of inflammation and immune system in the development of psoriasis (5, 6), the importance of biologic therapies targeting systemic inflammation (7), the significance of utilizing specific antibodies (8), the contribution of platelets to regulation of NET formation (9), the antimicrobial and pathogenetic roles of neutrophils in autoimmune, autoinflammatory, metabolic (10) and cardiovascular diseases (11). Also, recent books summarized the causes, symptoms, and treatment options of psoriasis (12–15).

**Figure 2:**
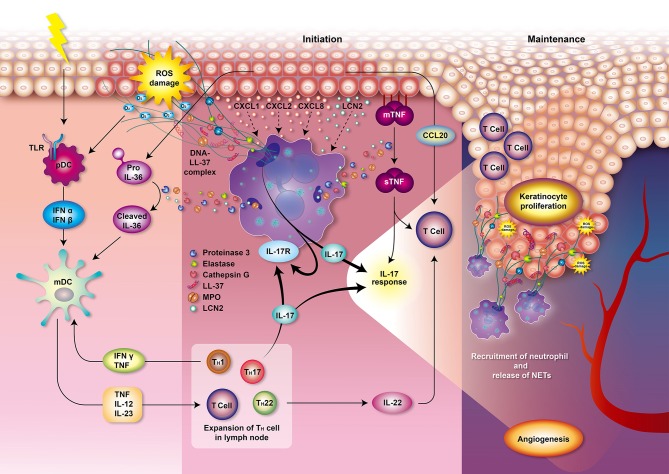
Role of neutrophils in psoriasis. Various and diverse endogenous and exogenous impulses such as antigens, trauma, infection, or emotional stress can trigger the complex immune reactions leading to psoriasis. The interplay of neutrophils, dendritic cells, and T cells bridges the innate immune and adaptive immune systems. T cells and keratinocytes release chemokines, such as IL-17, CXCL1, CXCL2, and CXCL8, that mediate the recruitment of neutrophils. Circulating neutrophils migrate to the psoriatic lesions and induce respiratory burst, degranulation, and formation of NETs, thereby contributing to the immunopathogenesis of psoriasis which involves T cell imbalance, keratinocyte proliferation, angiogenesis, and auto-antigen formation. Neutrophils from patients with psoriasis have enhanced NOX2 and MPO activity and augmented respiratory burst. MPO also participates in generating oxidative stress and upregulating degranulation. The accumulated oxidative stress produced by neutrophils could stimulate the antigen-presenting cells pDCs via TLR receptors 7 and 9 which stimulate antigen-specific CD8+ T cells (memory T cells in dermis and naive T cells in lymph node) to release cytokines, chemokines, and other innate immune mediators. These T cells may also migrate to epidermis and trigger local inflammation and keratinocyte proliferation via MHC I receptor of keratinocytes. Production of IFN-α and IFN-β by pDCs then stimulates mDCs to secrete pro-inflammatory mediators such as TNF, IL-12 and IL-23. Proteinase 3 released from neutrophils cleaves pro-IL-36 to activated IL-36 cytokine amplifying the response of mDCs. TNF, IL-12, and IL-23 play an important role in the initiation of the Th1, Th17, and Th22 cells immune response in lymph node, leading to a secretion of various cytokines and chemokines. IL-1 is further amplifying Th17 response while TNF and IFN-γ is creating a back loop to mDCs activation. Th17 activation then leads to the production of IL-17 activating neutrophils and keratinocytes via IL-17 receptors which generates profound IL-17 response. Keratinocytes produce TNF and CCL20, a chemotactic for T cell and DCs. Neutrophils degranulate and release MPO, NE, proteinase 3, cathepsin G and lipocalin. Proteinase 3 cleaves and converts the resting TNFα located in membrane of epithelial cells (mTNFα) to an activated state called soluble TNFα (sTNFα). Proteinase 3 also contributes to the formation of the LL-37 which serves as autoantigen. The chromatin of NETs (DNA) combined with LL-37 have a profound role in the initiation and maintenance of immune response in psoriasis. NETs further supply IL-17 and induce Th17 cells to release more IL-17, which plays a decisive role in the maintenance of psoriasis. These processes participate in the psoriasis complex inflammatory reactions and lead to the escalation of local psoriatic tissue inflammation. IL-22 contributes to the formation of characteristic psoriatic skin lesions including epidermal hyperplasia and acanthosis (thickening of skin). The activation of the following transcription factors promote TNF and IL-17 production and formation of downstream amplification loops in psoriasis: the Janus kinase (JAK)–signal transducer and activator of transcription (STAT) family, nuclear factor-κB (NF-κB) and cyclic AMP. Furthermore, the activation of endothelial cells induces vascular proliferation, angiogenesis and the expression of adhesion molecules in the endothelium to recruit additional inflammatory cells into the skin such as mast cells and macrophages contributing to the pathogenesis of psoriasis. CCL, CC-chemokine ligand; CXCL, chemokine (C-X-C motif) ligand; IL, interleukin; IFN, interfenon; MHC I, major histocompatibility complex class I, MPO, myeloperoxidase; NET, neutrophil extracellular trap; NOX, NADPH oxidase; NE, neutrophil elastase; pDCs, plasmacytoid dendritic cells; mDCs, myeloid dendritic cells; TLR, Toll-like receptor; TNF, tumor necrosis factor.

However, the role of neutrophils in psoriasis was not deeply analyzed and summarized based on recent literature. Thus, in this work, we focus on summarizing recent findings on the main offensive features of neutrophils including respiratory burst, degranulation and NETs and their direct connection with development and progression of psoriasis (Figure 2). We hope that our work would provide a foundation for further studies to attenuate overstimulation of neutrophils in psoriasis and aid patients with a debilitating disease.

## Psoriasis

Psoriasis affects ~2–3% of the world population (>125 million people). Psoriasis is a common, chronic, immune-mediated disease that is manifested mainly as skin lesions and extracutaneous comorbidities (16, 17). It is associated with systemic inflammation, similar to that observed in obesity, malignancy, psoriatic arthritis, cardiovascular disorders, chronic obstructive pulmonary diseases, type 2 diabetes mellitus, liver and renal diseases, and inflammatory bowel diseases (18–20). Psoriasis affects men and women equally and usually starts to be manifested at the age of 20 to 30, but children and teenagers can be also affected (21). Clinical types of psoriasis include psoriasis vulgaris, guttate psoriasis, inverse psoriasis, pustular psoriasis, and erythrodermic psoriasis (22). Typical skin manifestations of psoriasis include erythematous, indurated, and scaling plaques that are painful, itchy, and have a burning sensation (16, 23). Psoriasis decreases patients' quality of life due to unpleasant symptoms and related public stigma (24, 25). The unpleasant skin appearance contributes to reduced employment levels and thus, affects the financial status of patients (26). Depression and suicidal tendencies are also increased in patients with psoriasis (27, 28). Therefore, psoriasis results in long-term physical, psychological, and economic burden at both the individual and societal levels.

## Neutrophils in Psoriasis

Psoriasis is an immunogenetic disease that is associated with the interactions between the innate and adaptive immune systems (29). The immunology disturbance in psoriasis is related to overstimulation of neutrophils, dendritic cells, T cells, keratinocytes, fibroblasts (6, 30), mast cells (31), and melanocytes (30, 32). Munro's microabscesses filled with neutrophils, which were first described in 1898, are considered as one of the major histopathological hallmarks of psoriasis (33). Neutrophils are now thought to be regulators between the innate and adaptive immune systems (34, 35).

There is no cure for psoriasis, however the symptoms can be reduced either by avoiding triggers or by medications. Currently, the available treatments such as phototherapy, topical therapy (corticosteroids, vitamin D analogs), systemic therapy (methotrexate, apremilast, cyclosporin), and biological treatments offer a relieve for patients with different severity of psoriasis. However, the risk-benefit ratio must be well-considered on individual basis, particularly, considering the chronic course of the disease and limitations of the long-term use of certain drugs (16). Although T cell immunology-related treatments have emerged as attractive options for psoriasis, according to a systematic review of adherence and satisfaction to current treatment covering studies conducted in 2002 (36) or between beginning of 2009 and end of 2014 in European Union (37), psoriatic patients expressed only moderate satisfaction with the available treatments as evidenced by the poor adherence rates, in particular to topical treatments (36, 37). That might be the biggest motivating factor for the use of alternative treatment methods such as traditional Chinese medicine and herbs (*Scutellaria baicalensis, Zingiber officinale, Indigo naturalis, Mahonia aquifolium, Aloe vera*) (38), dietary supplements (fish oil, vitamin D) or other (39). Furthermore, the combination of certain alternative medicines with conventional drug therapies has been shown to improve the treatment efficacy, which points to the importance of evaluation of safety of combined treatments, education of doctors but also improvements in patient-doctor interactions (40). Indeed, modern biological therapies demonstrate improved safety and efficacy, as well as better satisfaction in patients, but belong to an expensive class of drugs, with limited availability (41). Biological therapies of psoriasis include monoclonal antibodies or inhibitors targeting tumor necrosis factor (TNF)α (infliximab, adalimumab; etanercept) (42–44), interleukin (IL)-23/IL-12 (ustekinumab) (42), IL-23 (guselkumab, tildrakizumab, risankizumab) (45, 46), or IL-17 (secukinumab, ixekizumab, brodalumab) (11, 47, 48). The available therapies indirectly affect the function and numbers of neutrophils. According to previous studies, the neutrophil-to-lymphocyte ratio (NLR) and platelet-to-lymphocyte ratio (PLR) are significantly increased in patients with psoriasis (49). While the elevated NLR and PLR values are particularly associated with psoriasis, they do not indicate the severity of the condition (50). The importance of NLR level in the progression of the disease was demonstrated by the reduction in NLR level following psoriasis treatment (51). That brings in question the effectiveness of certain treatments such as narrow-band ultraviolet B phototherapy which does not affect the increased level of NLR (52). Increased NLR and PLR levels emerged as unrecognized predictors of subclinical atherosclerosis in patients with psoriasis (53). The presence of excessive amount of neutrophils is characteristic for the generalized pustular psoriasis. Recent reports indicated that the depletion of neutrophils significantly relieves the symptoms of pustular psoriasis in patients that did not respond well to conventional treatments (54). Understanding the role of neutrophils in psoriasis attracted the attention of scientific community aiming to develop treatment protocols focusing on attenuating neutrophil overstimulation in this disease (55).

## Respiratory Burst and Psoriasis

Circulating neutrophils are recruited to inflammatory sites following inflammatory signals. They are then activated to generate and release large amounts of ROS in a phenomenon known as respiratory burst. NADPH oxidase (NOX2) and MPO are two key enzymes that contribute to the respiratory burst (56, 57). NOX2 is composed of transmembrane cytochrome b558 (p22^phox^ and gp91^phox^) and cytosolic subunits (p40^phox^, p47^phox^, p67^phox^, and Rac1/2). The assembled NOX2 complex, at the phagosomal and plasma membranes, is fully activated to generate superoxide anion (${\text{O}}_{2}^{∙-}$), which is the origin of various ROS produced by neutrophils. Superoxide (${\text{O}}_{2}^{∙-}$) is rapidly converted to hydrogen peroxide (H_2_O_2_) by superoxide dismutase (SOD). MPO, a heme peroxidase enzyme which is released in a process known as degranulation, utilizes H_2_O_2_ to produce many secondary reactive products. These products include hypochlorous acid (HOCl), chloramines (R-NHCl), and hypothiocyanite (OSCN–), as well as organic radicals such as products of lipid peroxidation (Figure 1) (58, 59).

ROS production is an integral part of the antimicrobial activity of neutrophils. However, the overproduction or inadequate clearance of ROS can cause various oxidative stress-related dysfunctions. These include cell and tissue damage; peroxidation and modification of DNA, lipids, and proteins; autoimmune NET formation; and autoantibody generation (58, 60). Neutrophils obtained from patients with psoriasis were shown to possess increased MPO and NOX2 activities, and release more ROS compared with neutrophils from healthy individuals (61, 62). Keratinocytes and T cells in psoriatic lesions produce priming agents of neutrophils, which results in an augmented respiratory burst by neutrophils with the overproduction of ROS (63–66). Accumulation of oxidative radicals also contributes to the pathogenesis of psoriasis. In response to the overproduction of ROS, dendritic cells are stimulated to present antigens to the T cells which results in an imbalance of T helper cell (Th)1 and Th2 cells, stimulation of keratinocytes proliferation, and promotion of angiogenesis (Figure 2). ROS then serve as the second messenger to activate mitogen-activated protein kinase (MAPK), nuclear factor-kappa B (NF-κB), or the Janus kinase-signal transducer and activator of transcription proteins (JAK-STAT)-related inflammatory pathways (67, 68). Suppression of respiratory burst of neutrophils emerged as a plausible pathway of attenuating overly immune response associated with psoriatic symptoms.

## Degranulation and Psoriasis

Neutrophils possess a multi-lobed nucleus, few mitochondria, and many specific storage organelles called granules. Granules are classified into azurophilic granules, specific granules, gelatinase granules, and secretory vesicles depending on their size, reaction with peroxidase-reactive dye, staining with 3,3'-diaminobenzidine, protein content, and tendency to mobilize (Figure 1) (69, 70). Azurophilic (peroxidase-positive or primary) granules are packed with MPO, bactericidal/permeability-increasing protein, defensins, lysozyme, and serine proteases, such as neutrophil elastase (NE), proteinase 3, and cathepsin G. Lysozyme is also found in specific and gelatinase granules (55). Specific (secondary) granules contain distinctive iron-binding glycoprotein lactoferrin, neutrophil gelatinase-associated lipocalin (NGAL, also called serum lipocalin-2, LCN2), collagenase, cytochrome b558, MAC-1 (CD11/CD18), and importantly, cathelicidins such as LL-37 (71). Cytochrome b558 and MAC-1 are also present in gelatinase granules and secretory vesicles (55). Gelatinase (tertiary) granules store gelatinase, lysozyme, arginase 1, and ficolin 1 (72). The secretory vesicles contain a characteristic alkaline phosphatase (57).

As activation processes of neutrophils are triggered by diverse stimuli such as bacterial or proinflammatory lipid mediators, neutrophils mobilize different granules and release the aforementioned granular components in a process known as degranulation or exocytosis (57). Degranulation is regulated by complicated control mechanisms, such as calcium signaling and actin remodeling (72–74). Azurophilic granules discharge toxic components into phagosomes and at inflammation sites. The secretion of specific and gelatinase granules promotes migration of neutrophils and the antimicrobial activity. The main purpose of releasing secretory vesicles is to facilitate neutrophils recruitment. Therefore, the degranulation process promotes firm adhesion, migration, respiratory burst, and successive NET formation of activated neutrophils (75). However, the dysregulation of neutrophil degranulation could damage tissues as observed in various diseases, such as hypoxia-related airway injury (76), severe pneumonia and chronic obstructive pulmonary diseases (77), atherosclerosis (78, 79), acute inflammatory liver injury (80), and rheumatoid arthritis (81, 82).

In psoriasis, MPO is significantly increased in skin plaques and is positively correlated with the severity of psoriasis (83). Serum MPO is also increased in patients with psoriasis, which may be related to recruited leukocytes in psoriatic skin lesions (84). MPO, the major enzymatic content of neutrophil granules, accounts for ~5% of the dry weight of the cell and represents the most toxic enzyme expressed by neutrophils (85). MPO is involved in the respiratory burst and can bind to CD11b/CD18 integrins, thereby contributing to the upregulation and augmentation of neutrophil degranulation in psoriasis (86). Furthermore, the neutrophil granule-derived serine proteinases, such as NE, proteinase 3, and cathepsin G, can activate interleukin (IL)-36 cytokine and lead to the escalation of local psoriatic tissue inflammation (87, 88). Proteinase 3 cleaves and converts the resting TNFα located in membrane of epithelial cells (mTNFα) to an activated state called soluble TNFα (sTNFα), which participates in the psoriasis complex inflammatory reactions (Figure 2). Proteinase 3 also contributes to the formation of the LL-37, an antimicrobial peptide belonging to cathelicidin family of polypeptides (89, 90), which serves as autoantigen mediating immune response in psoriasis (33). Antimicrobial peptides, synthesized by various leukocytes and epithelial cells, act via DNA/RNA complexes binding Toll-like receptors (TLR) 7, 8, and 9 to facilitate skin inflammation (91, 92). In addition, NE proteolytically activates the epidermal growth factor receptor (EGFR) signaling pathway resulting in excessive keratinocyte proliferation in psoriasis (93). Thus, the inhibition of neutrophils degranulation process or some of the enzymes contributing to psoriasis (NE, MPO, proteinase 3) are feasible targets for alleviating psoriatic symptoms.

## Nets and Psoriasis

The process of forming neutrophil extracellular traps (NETs) was first reported in 2004 (94). NETs are composed of extruded sticky chromatin ornamented with many antimicrobial components including histones, MPO, NE, cathepsin G, high mobility group protein B1 (HMGB1) and antimicrobial peptides, such as LL-37 (Figure 1) (95). NETs can catch and destroy pathogens in order to prevent microbes from spreading (96). However, the dysregulated formation and clearance of NETs can result in many diseases. These include autoimmune diseases, such as systemic lupus erythematosus, anti-neutrophil cytoplasmic autoantibody (ANCA)-associated vasculitis, rheumatoid arthritis, gout (97), and autoimmune hepatitis (98); cardiovascular diseases, such as atherosclerosis, thrombosis, and abdominal aortic aneurysm (99–101); respiratory disorders inclusive of asthma, chronic obstructive pulmonary disease, cystic fibrosis, tuberculosis, bacterial and viral pneumonia, and transfusion-related acute lung injury (102, 103); digestive diseases, such as inflammatory bowel diseases, primary sclerosing cholangitis, primary biliary cholangitis (98); and cancer-related organ damage, metastasis, and thrombosis (9). Recently, a role of NETs in awakening of dormant cancer cells was discovered (104).

The process of NET formation is termed NETosis, which is subdivided into lytic NETosis and non-lytic NETosis. In lytic NETosis (also called suicidal NETosis), activated neutrophils generate NETs (it takes 2–4 h) and undergo a programmed cell death, which differs from necrosis, necroptosis, and apoptosis (105). Lytic NET formation is triggered by various stimuli undergoing different pathways. For instance, phorbol myristate acetate (PMA) increases cytosol calcium, activates protein kinase C (PKC)/Raf/MEK/ERK pathway, and induces NOX2 to generate ROS. ROS then acts as the second messenger to disintegrate the nuclear membrane and stimulate MPO to translocate NE to the nucleus where it causes proteolysis of histones and decondensation of chromatin. Afterward, peptidyl arginine deiminase 4 (PAD4)-mediated hypercitrullination of histones allows decondensed chromatin, the main component of NETs, to be readily expelled from the cell nucleus. Finally, as the plasma membrane dissolves, the chromatin decorated with granular components is released as extracellular traps (106). Other pathways of lytic NETosis include stimulation by fungi (such as *Aspergillus* spp.) through Dectin 2 and complement receptor 3 (CR3) (107), LPS (lipopolysaccharide) under special conditions described later in detail (108) or by monosodium urate crystals via receptor-interacting serine/threonine-protein kinase 1 (RIPK1) and RIPK3 pathway (109). All of these pathways involve NOX2, MPO, and NE activation (105). Nevertheless, there are other stimulators of NETosis acting independently of NOX2 such as ionomycin, or immune complexes (110). Ionomycin induces NETs via small conductance calcium-activated potassium channel protein 3 (SK3) and protein kinase C ζ (PKCζ), mitochondrial ROS (mitoROS), NE, and protein-arginine deiminase type 4 (PAD4) (111). Immune complexes related NETosis through FcγRIIIb are highly dependent on mitoROS (110, 112). The lytic-NETosis inducers such as PMA, ionomycin, or living bacteria were confirmed using a live imaging confocal microscopy, however, dead bacteria, LPS, glucose, or activated platelets alone failed to induce NETosis in the *in vitro* experiment (113). Such discrepancy might be due to variations in the experimental design of various studies.

On the other hand, non-lytic NETosis (also called vital NETosis) does not require neutrophils lysis or even the breach of the plasma membrane. Following the release of NETs, neutrophils are alive and keep their functions, such as chemotactic movement, phagocytotic ability, and respiratory burst power (98). This form of NETosis usually occurs early in infection by Gram-positive bacteria in human and mice. The process is very rapid (5–60 min to form NETs), requires both TLR 2 and complement-mediated opsonization, and is independent of NOX2 (114). Non-lytic NETosis can be induced by *Staphylococcus aureus* via a unique mechanism where the inner and outer nuclear membranes are separated, and the vesicles filled with nuclear DNA are extruded intact into the extracellular space where they rupture and release chromatins. Despite that this type of NETs keep a limited amount of proteolytic activity it is still able to kill *S. aureus* (115). Non-lytic NETosis can also be stimulated by *Candida albicans* via interaction with CR3 and fibronectin (116). Moreover, a special type of non-lytic NETosis, which releases mitochondrial DNA and is dependent on ROS, is stimulated by the granulocyte-macrophage colony-stimulating factor (GM-CSF) and LPS (10). Interestingly, Leishmania parasites induce both lytic and non-lytic NETosis (117). In that case, the chromatin decondensed by PAD4 is mixed with granular proteins and subsequently excreted with a minor nuclear envelope disruption and without cell membrane disorganization (10, 98). Delgado-Rizo et al. previously summarized the microbial inducers of NETs (10) but we would like to clarify the effect of LPS. Lipopolysaccharide (LPS) is an important component of the outer membrane of gram-negative bacteria known to trigger immune response (118). For a long time, it was unclear whether the direct interaction between LPS and neutrophils causes NETs release, because several reports showed LPS-induced lytic NET formation (94, 119) while other not (113, 120). Recently, it has been shown that only species- and serotype-specific LPS is able to induce NETs by direct interaction with neutrophils. It was demonstrated that LPS has to be derived from specific bacterial strain of *Escherichia coli* (O128:B12) and *Pseudomonas aeruginosa* (serotype 10) and must be present at sufficient concentration (8 pg per neutrophil). The neutrophils then undergo a lytic-NETosis independent of TLR4. However, non-lytic NETosis is triggered when sufficient amount of LPS regardless of bacterial origin interacts with TLR4 of platelets (108). The process is followed by binding of platelets to the P-selectin glycoprotein ligand-1 (PSGL-1) of neutrophils, and the release of HMGB1 by platelets (9, 95). Moreover, there is a growing evidence of crucial role of the other endogenous and immune factors in the process of NET formation, such as presence of platelets (95, 120), glucose (10), or other effectors (121). To orchestrate inflammatory response, NETs in combination with LPS were shown to induce the production of IL-1β by J774 macrophages via the caspase-1 and caspase-8 pathways (122).

In patients with psoriasis, neutrophils are pre-activated and form NETs in psoriatic skin lesions (55, 123). NETs are increased in blood samples and correlate with the severity of psoriasis (124, 125). They create an extremely immunogenic environment and participate in the initial and maintenance phases of psoriasis (126, 127). NETs stimulate epidermis to release inflammatory cytokines via TLR4 and IL-36 receptor crosstalk (123). Various exogenous and endogenous stimuli and ROS generated by neutrophils initiate immune reaction leading to psoriasis which involves T cell imbalance, keratinocyte proliferation, angiogenesis, and auto-antigen formation (Figure 2). The chromatin of NETs in psoriasis plaques is accompanied with antimicrobial peptide LL-37 released by keratinocytes to stimulate the synthesis of inflammatory mediators including IFN-α and IFN-β in plasmacytoid dendritic cells (pDCs) (16). Myeloid DCs (mDCs) are then activated to release many pro-inflammatory mediators, such as IL-6, IL-12, IL-23, and TNFα (91, 92), which play an important role in the initiation of the Th1, Th17, and Th22 cells immune response (16). Proteinase 3 released from neutrophils cleaves pro-IL-36 to activated IL-36 cytokine which is together with TNF and IFN-γ amplifying the response of mDCs. Th17 activation then leads to the production of IL-17 activating neutrophils and keratinocytes via IL-17 receptors which generates profound IL-17 response (16). Secretory leukocyte protease inhibitor (SLPI), a component of NETs with an inhibitory function on NET formation, may bind to DNA and NE in psoriatic skin lesions and activate the pDCs to produce type 1 interferons (IFN-α, IFN-β, etc.) which regulates autoimmunity in psoriasis (128–130). In addition, NETs allow the mDCs to readily sense the neutrophilic antigens and allow the T cells to be primed directly (45, 131, 132). Thus, NETs play an important role in the pathophysiology of psoriasis due to their link between the innate and adaptive immune systems. Psoriasis is accompanied with increased serum levels of TNF-α, interferon (IFN)-γ, IL-1, IL-2, IL-4, IL-6, IL-8, IL-10, IL-12, IL-17, IL-18 (133), IL-22 (134), chemerin, resistin (135), lipocalin-2 (LCN2) (123), soluble E-selectin (sE-selectin) (136), complement 3 (137), and decreased levels of transforming growth factor-beta (TGF-β) and adiponectin (133). These cytokines may therefore serve as potential biomarkers for psoriasis and treatment response in patients. According to a cross-sectional study, psoriasis patients had increased proinflammatory macrophage type 1 (IL-1, IL-6, TNF-α), Th1 (IL-2, IL-12, IFN-γ), Th17 (IL-6, IL-17) but also anti-inflammatory Th2/T regulatory (Treg) (IL-4, IL-10) profiles which may be correlated to the severity of psoriasis (133). Among the important mediators in psoriasis, LCN2 acts as an antimicrobial protein as well as adipokine associated with obesity, insulin resistance, and atherosclerotic disease, and is also responsible for the activation of the immune system in response to inflammatory and toxic stimuli. Importantly, serum LCN2 levels are elevated in psoriatic patients (138) and correlate with the severity of itching and thus might be used as a clinical marker for itching in psoriasis (139). Not only granulocytes but also keratinocytes of epidermis secrete LCN2, which drives the chemotaxis of neutrophils and sustains NET formation, and thereby in turn maintains the psoriatic inflammation (123). The increased LCN2 blood levels were observed in patients with palmoplantar pustular psoriasis (140) as well as other chronic inflammatory skin diseases such as acne inversa (141) or atopic dermatitis (142). Wolk et al. reported a positive correlation between the LCN2 production and IL-1β levels in the epidermis, which was further enhanced by IL-17 and TNF-α, but not by IL-22. The contribution of LCN2 on skin neutrophil infiltration is apparent (141). In the clinical setting, tissue LCN2 was found to be also significantly higher in psoriasis, regardless of dyslipidemia, or metabolic disturbance in patients. But the LCN2 levels together with psoriasis area and severity index (PASI) score significantly dropped after NBUVB treatment (143). The *in vivo* effects of LCN2 on topical imiquimod (IMQ)-induced psoriasis-like skin in BALB/c mice were evaluated by Hau group (138). In addition to markedly exacerbated erythema and scaling in IMQ-treated murine skin, LCN2 increased the mRNA expression of interleukin (IL)-17A, IL-17F, IL-22, IL-23, CC-chemokine ligand (CCL)20, TNF-α, chemokine (C-X-C motif) ligand (CXCL)1, CXCL2, LCN2, and S100A7 while it did not affect the mRNA levels of IFN-γ, or CXCL10 in the skin. Similar effects were observed *in vitro* on human keratinocytes (138). The data suggest a link between NETs-related cytokines and Th-17 activation in psoriasis.

## Net-TH17 Axis and Psoriasis

Psoriasis has been considered as a T-helper (Th)1/Th17-mediated, chronic inflammatory dermatosis with relation to metabolic syndromes (144). Apart from keratinocytes and T lymphocytes (145), neutrophils are one of the major cellular sources of IL-17 via NET formation in psoriasis (146), and also mast cells were reported to generate extracellular traps (147). NETs activation has been linked with Th17 responses in psoriasis and has drawn particular attention recently (148). In an experimental model, NETs induced the generation of CD3^+^CD4^+^IL-17^+^ (Th17) cells from peripheral blood mononuclear cells, which requires monocyte and cell-to-cell contact. Th17 induction was enhanced by a psoriasis risk-associated variant in the TRAF3IP2 gene encoding the D10N variant of Act1 which serves as a key mediator of IL-17 signal transduction. That provides an evidence of genetic basis for the enhanced IL-17 expression in psoriasis. IL-17-expressing T cells and neutrophils were suggested to have a cross-talk because IL-17-expressing T cells produce cytokines which promote the development, recruitment, and lifespan of neutrophils (149).

Although many immune diseases including psoriasis or atopic dermatitis have been traditionally classified as Th1/Th2 biphasic disorders, there is a growing evidence supporting a rather systemic activation of other multiple Th-cell subsets, such as Th17 cells producing IL-17 and IL-22 (Figure 2). Interestingly, in comparison with psoriasis, atopic dermatitis showed reduced genomic expression of IL-23, IL-17, IFN-γ, and other innate defense genes (hBD2, elafin, LL-37) (150). Elevated IL-17 is detected in psoriatic skin lesions and in the blood (151). IL-17 mainly stimulates keratinocytes to produce neutrophil-tropic chemokines CXCL-1, CXCL-2, CXCL-8 (IL-8), and antimicrobial peptides, such as LL-37. IL-17 serves as an autocrine-amplifying mediator that is simultaneously involved in the recruitment, activation, and survival of neutrophils (6). There are several subtypes of IL-17 family cytokines binding to IL-17 receptors, namely IL-17A, IL-17B, IL-17C, IL-17D, IL-17E (also known as IL-25) and IL-17F (145). IL-17A from neutrophils stimulates keratinocytes to express CCL20, attracting Th17 cells with CCR6 expression to release IL-17A, and finally resulting in positive feedback and the development of the psoriatic lesions (8). IL-17C is a unique cytokine that is produced by keratinocytes and that is involved in such synergistic loops that may be responsible for amplifying the inflammation in both psoriasis and atopic dermatitis. This may ultimately lead to induction of S100As and other molecules that accompany epidermal hyperplasia. Thus, antagonism of IL-17C may be beneficial for psoriasis and atopic dermatitis treatment (152). IL-17E then recruits neutrophils by activating macrophages and contributes to the infiltration of psoriatic neutrophils (153). Besides other innate immune cells, neutrophils significantly contribute to IL-17-related immune regulations in psoriasis by employing several mechanisms including the formation of NETs (45). Moreover, IL-17 released by NETs leads to endothelial dysfunction in atherosclerosis and keratinocyte proliferation in psoriasis, which may explain why patients with psoriasis also suffer from increased risk of atherosclerosis (11). Anti-IL-17 drugs, such as secukinumab, clear the neutrophils in the epidermis and can be used to effectively treat psoriasis (48). The targeted biologic therapies are of great importance with regards to an increasing number of comorbidities associated with psoriasis together with its systemic inflammation nature indicating that these diseases are sharing some common pathological mechanisms (7). In summary, NETs were demonstrated as potential upstream drug targets for the treatment of psoriasis.

## Conclusion

Neutrophils in psoriasis are of interest, particularly, because of their crucial roles in the innate and adaptive immune system. The respiratory burst with ROS generation, degranulation, and formation of NETs are the main offensive functions of neutrophils and contribute to the immunopathogenesis of psoriasis. Recently, great attention was brought to the role of NETs in psoriasis because activated neutrophils producing NETs are abundant in psoriatic skin plaques and pustules, as well as in the serum of patients with psoriasis. Overexpression of NETs leads to the activation of other cells releasing IL-17, which stimulates the synthesis of inflammatory mediators and in turn leads to auto-amplification of neutrophils. Therefore, further development of inhibitors and biologic drugs targeting overexpressed offensive features of neutrophils, i.e., respiratory burst, degranulation, and NET formation, is of great importance. We believe that the consideration of the role of neutrophil defense mechanisms in the pathogenesis of psoriasis offered in this review highlights the need to further investigate neutrophils for possible improvements of available treatments in the future.

## Author Contributions

C-CC and W-JC wrote and revised the manuscript. MK consulted and revised the manuscript. C-YL drew the figures. T-LH initiated the concept and supervised the writing. All authors read and approved the final manuscript.

### Conflict of Interest

The authors declare that the research was conducted in the absence of any commercial or financial relationships that could be construed as a potential conflict of interest.

## Abbreviations

CCL: CC-chemokine ligand
CXCL: chemokine (C-X-C motif) ligand
IL: interleukin
IFN: interfenon
LPS: lipopolysaccharide
mDCs: myeloid dendritic cells
MPO: myeloperoxidase
NET: neutrophil extracellular trap
NOX: NADPH oxidase
NE: neutrophil elastase
NLR, neutrophil-to-lymphocyte ratio, pDCs: plasmacytoid dendritic cells
PLR: platelet-to-lymphocyte ratio
Th: T helper cell
TLR: Toll-like receptor
TNF: tumor necrosis factor.
